# Recognition of stroke symptoms indicative of anterior circulation large-vessel occlusion via telephone and video calls: a simulation study

**DOI:** 10.1186/s12873-025-01344-3

**Published:** 2025-09-10

**Authors:** Matthias L. Herrmann, Simone Meier, Florian F. Schuchardt, Max Henningsen, Nicole Wimmesberger, Diana Rau, Erik Farin-Glattacker, Jochen Brich

**Affiliations:** 1 Department of Neurology and Clinical Neuroscience, Faculty of Medicine and Medical Center, University of Freiburg, Freiburg, Germany; 2 Section of Health Care Research and Rehabilitation Research (SEVERA), Institute of Medical Biometry and Statistics, Faculty of Medicine and Medical Center, University of Freiburg, Freiburg, Germany; 3 Department of Neurology and Clinical Neuroscience, Freiburg University Medical Center, Breisacher Str. 64, 79106 Freiburg, Germany

**Keywords:** Emergency medical dispatch, Stroke, Emergency control center, Large vessel occlusion, Prehospital triage, Video livestreaming

## Abstract

**Background:**

Identifying suspected anterior circulation large-vessel occlusion (aLVO) strokes during emergency calls could enhance dispatch efficiency, particularly in rural areas. However, data on emergency medical dispatchers’ (EMDs) ability to recognize aLVO symptoms remain limited. This simulation study aimed to evaluate the feasibility of identifying side-specific arm paresis, side-specific conjugate eye deviation (CED), and aphasia during emergency calls by instructing layperson callers to perform brief, standardized examination steps. Two communication methods were compared: (1) telephone calls and (2) video calls.

**Methods:**

Forty-eight laypersons interacted with simulated patients presenting various stroke syndromes. Simulated EMDs conducted standardized assessments during simulated emergency calls, guiding laypersons through patient examinations.

**Results:**

In 96 telephone-assisted and 95 video-assisted calls, EMDs identified aLVO stroke symptoms with high accuracy. In telephone calls, accuracy was 0.92 for side-specific arm paresis, 0.98 for side-specific CED, and 0.88–0.99 for aphasia. In video calls, accuracy was 0.97 for side-specific arm paresis, 0.97 for side-specific CED, and 0.94-1.00 for aphasia.

**Conclusions:**

These findings suggest that EMDs can identify stroke symptoms indicative for aLVO via both telephone and video calls using a standardized dispatch protocol to guide lay bystanders. This study provides a foundation for future real-world research on implementing aLVO detection protocols in emergency dispatch.

**Supplementary Information:**

The online version contains supplementary material available at 10.1186/s12873-025-01344-3.

## Background

The identification of patients with suspected anterior circulation large-vessel occlusion (aLVO) stroke by emergency medical dispatchers (EMDs) during emergency calls has the potential to reduce time to endovascular thrombectomy by enabling optimized dispatch strategies. For instance, in rural areas, parallel dispatching of helicopter emergency medical services can help mitigate prolonged ground transport times to the nearest comprehensive stroke centers [1]. However, telephone stroke detection by EMDs is error-prone and the overall accuracy of detection is relatively low, even when using structured questionnaires [2–4]. These typically include screening questions on the presence of any arm weakness, facial asymmetry, and speech difficulties—an undifferentiated combination of dysarthria and aphasia—based on the Face Arm Speech Time (FAST) test [5].

Currently, no data are available on EMDs’ ability to reliably identify aphasia (distinct from dysarthria) or neglect, which are critical cortical signs known to be strong predictors of aLVO stroke [6]. Limited data on EMDs’ ability to detect conjugate eye deviation (CED), a component of the neglect syndrome and another highly predictive cortical sign of aLVO stroke [6], have yielded mixed results [7].

A promising method for detecting visually recognizable stroke symptoms, such as CED or hemiparesis, is the use of live video streams from the caller’s smartphone to the emergency call center (ECC). Video calls (VCs) could also enhance the identification of aphasia (as distinct from dysarthria) by enabling direct verbal interaction with the patient under visual observation. In recent years, a growing number of studies have explored the use of VCs, predominantly in the context of emergency medical dispatcher (EMD)-assisted cardiopulmonary resuscitation [8, 9]. However, the application of VCs for stroke symptom recognition by EMDs has not yet been studied [9].

Given the rarity of aLVO stroke cases in routine EMD practice [10], and the time-critical nature of emergency calls, a simulation study offers a practical approach to evaluate the feasibility of detecting symptoms indicative of an aLVO stroke. Simulated patients (SPs) who have undergone intensive training have been shown to accurately and convincingly replicate stroke symptoms and syndromes [11], providing a realistic and controlled environment for such research.

The primary objective of this simulation study was to assess the feasibility of identifying cortical symptoms indicative of aLVO stroke during an emergency call—in which stroke has already been identified—by guiding the lay caller through simple, standardized examination steps. These steps targeted three key stroke symptoms: side-specific arm paresis, side-specific CED, and aphasia. Two different communication modalities were employed and compared: (1) telephone and (2) video call.

## Methods

### Study design

This simulation study was conducted at Freiburg University Hospital in September 2020, following approval by the Ethics Committee of the University of Freiburg (Application number: 416/20). Written informed consent was obtained from all participants prior to their inclusion in the study.

### Simulated patients

Eight simulated patients (SPs) were included, each extensively trained by experienced neurologists to accurately and convincingly simulate stroke symptoms [11]. Each SP represented a specific stroke syndrome commonly observed in patients with acute anterior circulation stroke (Supplementary Table 1).

### Lay bystanders and simulation scenario

A total of 48 laypersons were recruited through public advertisements. Inclusion criteria required participants to be aged ≥ 18 years, have no prior professional medical experience, and possess sufficient proficiency in the German language. Laypersons received a brief introduction to the scenario: they were informed they would sequentially enter four rooms, each containing a person simulating a stroke, but were not provided details about specific stroke symptoms. In each room, laypersons used a provided smartphone to make a simulated emergency call to a predesignated number. Each participant completed two telephone calls and two video calls (VCs) in a random order determined beforehand, using Skype^®^ software. Participants were unaware in advance whether they would conduct a telephone call or VC. In the case of VC, communication began with a phone call, then the simulated emergency medical dispatchers (SEMD) instructed participants to activate the video camera. A study team member attended each session to address potential technical issues.

### Simulated emergency medical dispatchers

The study included 24 simulated emergency medical dispatchers (SEMDs), comprising four EMDs, seven paramedics, and 13 nurses. SEMDs were stationed in rooms adjacent to the SPs. Each SEMD handled four telephone calls and four VCs, with different laypersons evaluating different SPs during each call. SEMDs were blinded to the specific stroke symptoms being simulated.

### Standardized dispatch protocol for telephone calls

In collaboration with four experienced EMDs from the Freiburg ECC, a standardized dispatch protocol was developed. The protocol provided lay-friendly, step-by-step instructions to assess three stroke symptoms: side-specific arm paresis, side-specific CED, and aphasia. To ensure clarity, assessments relied on dichotomous responses rather than symptom severity scores.

**Fig. 1:**
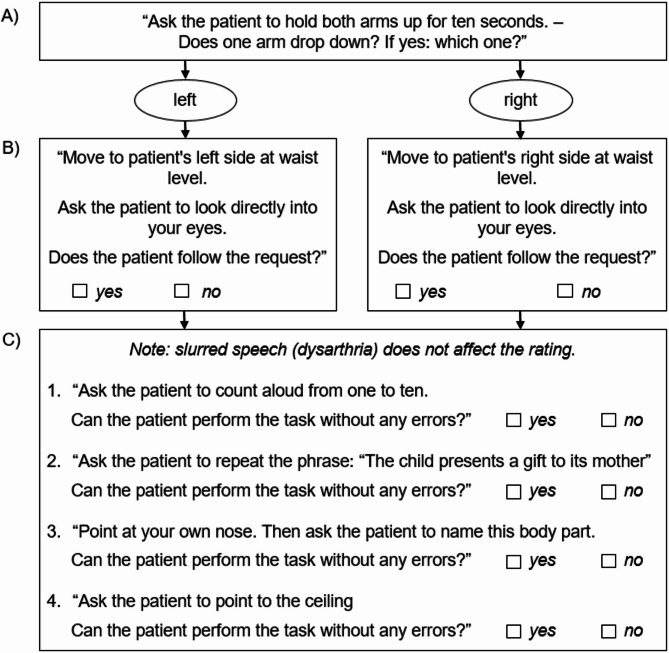
Standardized protocol for SEMDs for sequential telephone-based detection of the side of arm paresis, CED, and aphasia. The SEMDs asked a series of questions in a specific order to identify (**A**) arm paresis and if so, the side of the arm paresis, (**B**) side-specific CED (inability of SPs to turn their gaze to the side of the paresis), and (**C**) aphasia, based on four different tasks covering different domains of aphasia: automatic speech (**C-1**), repetition (**C-2**), naming (**C-3**), and comprehension (**C-4**)

- Arm Paresis: The SEMD instructed the caller to ask the SP to raise both arms for 10 s (Fig. 1, Step A). The caller reported if one arm dropped and, if so, which arm.
- CED: To ensure a standardized assessment of corresponding CED in opposite to the arm paresis, the SEMD instructed the caller to stand next to the SP on the side of the arm paresis and observe whether the SP could direct their gaze towards the side of the paresis (Fig. 1, Step B).
- Aphasia: Four tasks were developed together with experienced EMDs to evaluate automatic speech, repetition, naming, and comprehension (Fig. 1, Step C). The automatic speech and the comprehension tasks were based on the Language Screening Test (LAST), which has previously been validated in patients with acute ischemic stroke [12, 13]. The repetition test was derived from the Bielefeld Aphasia Screening [14], a German language screening tool for the assessment of acute aphasia. To ensure the feasibility of the naming task at any location and without equipment, we chose to name the caller’s nose as an object that is always present in the emergency situation. For consistency, callers were instructed to score the first response and refrain from assisting the SP with verbal or nonverbal cues. Due to complexity, two attempts were permitted only for the repetition task. For all aphasia tests, the caller was instructed to focus strictly on the content and not on the articulation of the SP.

### Standardized dispatch protocol for video calls

For VCs, laypersons were instructed to activate the smartphone’s front camera and ensure that the SP’s arms and eyes were clearly visible. SEMDs provided identical instructions to those in the telephone protocol but slightly adapted for the video format. Unlike telephone calls, SEMDs directly interacted with the SPs and evaluated responses in real time (Supplementary Fig. 1).

### Statistical analysis

Accuracy, sensitivity, and specificity were calculated for each task within the standardized dispatch protocol for both telephone and video calls. Differences in symptom recognition accuracy between telephone and VC modalities were assessed using Fisher’s exact test. Statistical analyses were performed using IBM SPSS Statistics, version 28 (IBM Corporation, Armonk, NY, USA), with a significance threshold set at *p* < 0.05.

## Results

### Layperson characteristics

Sociodemographic data were available for 46 of the 48 laypersons (95.8%). The median age was 37.5 years (range: 18–84 years, interquartile range: 25–62 years). Of the participants, 31 (67%) were female, and 19 (40%) had a college education. 18 laypersons (39.1%) had either a relative or friend who had suffered a stroke in the past.

### Recognition of stroke symptoms by SEMDs using telephone calls

SEMDs responded to 96 simulated telephone emergency calls. They correctly identified side-specific arm paresis in 88 of 96 cases (91.7%), with correct identification rates of 95.7% (45/47) for right-sided paresis and 87.8% (43/49) for left-sided paresis. The presence of corresponding conjugate eye deviation (CED) was correctly identified in 29 of 31 cases (93.5%), and its absence was correctly determined in all cases (57/57; 100%). Regarding aphasia, the SEMDs correctly scored the automated speech test in 23/23 (100%) calls in which SPs simulated aphasia and in 70/73 (95.9%) calls in which SPs did not have aphasia. The repetition test was correctly scored in 23/23 (100%) cases with aphasia and in 69/73 (94.5%) cases without aphasia. The naming test was correctly scored in 18/23 (78.3%) cases with aphasia and in 66/73 (90.4%) cases without aphasia. The comprehension test was correctly scored by the SEMDs in 95/96 (99.0%) calls. Due to the study design, all SPs were able to perform the comprehension task correctly. Therefore, it was not possible to calculate sensitivity for this test (Table 1).

### Recognition of stroke symptoms by SEMDs using video calls

SEMDs responded to 95 simulated video-assisted emergency calls (one VC was canceled due to technical issues). They correctly identified side-specific arm paresis in 92 of 95 cases (96.8%), with correct identification rates of 98.0% (48/49) for right-sided paresis and 95.7% (44/46) for left-sided paresis. For CED detection, SEMDs identified its presence in 94.3% (33/35) of cases and its absence in 96.6% (57/59) of cases. Regarding aphasia, SEMDs correctly rated the automatic speech test in 25/25 (100%) calls in SPs simulating aphasia, and in 70/70 (100%) calls in which SPs had no aphasia. The repetition test was correctly scored in 25/25 (100%) cases with aphasia, and in 69/70 (98.6%) cases without aphasia. The naming test was correctly scored in 23/25 (92.0%) cases with aphasia, and in 66/70 (94.3%) cases without aphasia. The comprehension test was correctly rated by the SEMDs in 95/95 (100%) calls.

**Table 1:** Identification of side of arm paresis, CED and aphasia by SEMDs using a standardized algorithm

Symptom	Telephone				Video call				*p*
	*N*	ACC	SEN	SPE	*n*	ACC	SEN	SPE	
Arm paresis left	49	0.92	0.88	0.96	46	0.97	0.96	0.98	0.270
Arm paresis right	47	0.92	0.96	0.88	49	0.97	0.98	0.96	0.613
CED	88*	0.98	0.94	1.00	92*	0.97	0.97	0.97	1.000
Automatic speech	96	0.97	1.00	0.96	95	1.00	1.00	1.00	0.246
Repetition	96	0.96	1.00	0.95	95	0.99	1.00	0.99	0.368
Naming	96	0.88	0.78	0.90	95	0.94	0.92	0.94	0.215
Comprehension	96	0.99	-	0.99	95	1.00	-	1.00	1.000

ACC: Accuracy. SEN: Sensitivity. SPE: Specificity. CED: Conjugated eye deviation. *To avoid subsequent errors, 11 cases with incorrect detection of paresis (telephone: *n* = 8, video call: *n* = 3) were excluded due to the dependence of the correct identification of CED on the side of the paresis

No statistically significant differences in symptom recognition accuracy were observed between video and telephone calls (Table 1, column “p”).

## Discussion

This simulation study suggests that stroke symptoms, including “side-specific arm paresis,” “side-specific CED,” and “aphasia,” can be effectively identified in both telephone and video-assisted emergency calls when a standardized protocol is used to guide layperson callers. These findings form the basis for the effective detection of large vessel occlusion (aLVO) strokes by EMDs.

Both methods provided highly accurate results for identifying arm paresis, which aligns with previous simulation studies on telephone emergency calls [15, 16]. Video calls, while showing slightly higher accuracy, did not significantly outperform telephone calls. The ability to reliably identify the side of the paresis might enable the use of a side-specific sequential stroke severity scale for further specific cortical sign evaluation, which can streamline the diagnostic process and reduce assessment time—an essential requirement for emergency stroke dispatch protocols.

In our study, SEMDs demonstrated excellent accuracy in assessing CED using both telephone and video calls. These findings differ from a recent study where asking about CED was not highly predictive for identifying aLVO during telephone-based assessments under real-life conditions [7]. In that study, EMDs relied on a binary question to determine whether the patient’s face or gaze was turned away from the side of the hemiparesis. This approach may be prone to errors in uncontrolled emergency situations (e.g., when stimulation is provided from only one side or the patient’s position naturally favors a certain gaze direction). To address these potential confounders, we instructed callers to position themselves on the patient’s paretic side, which may have significantly improved the accuracy of their observations in our study. Consequently, both telephone and video call interpretations achieved near-perfect accuracy in our controlled setting. In real-world scenarios, it could be speculated that visual inspection of this distinct sign may be easier for EMDs via video than interpreting descriptions provided over the phone.

For the reliable detection of aphasia, a structured protocol incorporating four simple, validated tests—automatic speech, repetition, naming, and comprehension—was employed to ensure comprehensive assessment across core language domains. Emphasizing speech content over articulation enabled effective differentiation between aphasia and dysarthria. All tests proved suitable for use by layperson callers under the guidance of SEMDs, with clear instructions and no reliance on visual aids, facilitating application in telephone-based settings. The overall recognition accuracy did not differ significantly between telephone and video calls. There was a slight trend toward video calls in the naming test. This could reflect the advantage of direct communication between SEMDs and SPs, which avoids errors in the transfer of information. In contrast to prior approaches that combine aphasia and dysarthria detection for general stroke recognition (e.g., Cincinnati Prehospital Stroke Scale or the FAST test [3, 15, 16]), the current approach focuses on aphasia only as a cortical sign, that is strongly indicative of aLVO stroke [6].

In our simulation study, there were overall only minimal differences in the accuracy of SEMDs’ identification of stroke symptoms between the two query methods. This lack of variation may be attributable to the high overall performance achieved with both approaches, potentially leading to a ceiling effect that obscured any measurable differences. In real-world settings, video calls (VCs) could complement traditional audio-based emergency calls by aiding dispatchers in stroke detection, particularly for visually identifiable cortical symptoms commonly associated with aLVO, such as the combination of hemiparesis and CED or neglect. Additionally, the assessment of verbal symptoms in patients with aphasia might benefit from direct visual interaction via VC between the EMD and the patient through the caller’s smartphone.

A recent scoping review [9] of 24 studies highlighted overwhelmingly positive attitudes among EMDs toward VCs. The review noted that EMDs found VCs reassuring for both themselves and callers, easy to use, and beneficial for improving decision-making and patient assessment accuracy. However, practical evidence for the use of VCs to detect stroke symptoms, particularly cortical signs suggestive of aLVO, is still lacking.

The findings from this simulation study lay the groundwork for developing a standardized aLVO query for EMDs, which will be further evaluated in the upcoming LESTOR trial [17].

### Limitations

This study has several limitations. First, the simulation design restricts the generalizability of our findings. To ensure realistic scenarios, we employed specially trained standardized patients (SPs) proven to accurately and convincingly simulate acute stroke symptoms and syndromes [11]. However, under real-life conditions, EMDs may encounter additional challenges when using the query. Lay bystanders - particularly relatives or spouses - may experience heightened stress during the emergency call, which could affect their patience and reliability compared to our study participants. Moreover, patients themselves may be uncooperative, especially if they have aphasia with impaired comprehension (a condition excluded in our study design), complicating their ability to follow EMD instructions. In such cases, video calls (VCs) could provide advantages, allowing rapid visual detection of stroke symptoms. Additionally, patients may not always be supine, as assumed in this study, and may require repositioning. Language barriers may also hinder the execution of examination steps. While such barriers complicate the entire emergency call rather than specifically an anterior circulation large vessel occlusion (aLVO) stroke query, advances in artificial intelligence may eventually offer real-time translation solutions.

Second, although EMDs and callers were blinded to the SPs’ specific symptoms, they were aware that they would encounter SPs with stroke symptoms during the simulated calls. In practice, considering stroke as the first step in the dispatch process is often difficult and error-prone, even when using standardized stroke screening tools such as FAST. Moreover, since the prevalence of the stroke symptoms differ greatly in our simulation study (all simulated patients exhibit stroke symptoms) from the real EMD setting (where a call concerning stroke is relatively rare, and aLVO even rarer), the results may be misleading if taken to represent how performance would be in a non-simulated setting, since this important difference in prevalence will invariably impact the reliability of the results as pertains to the real-life setting.

Third, our study focused on the ability of EMDs to correctly identify the side of arm paresis. Consequently, all SPs presented with either right- or left-sided arm paresis, preventing us from assessing EMDs’ ability to identify the absence of arm paresis.

Fourth, we did not measure the exact duration of each encounter, although a time limit of 10 min per call was enforced in our study settings.

Finally, the median age of the laypersons in our study was 37.5 years, which may not accurately represent the broader population of emergency callers. Additionally, 67% of the participants were female, which likely does not reflect the gender distribution of callers. 39% of the laypeople reported having experienced a stroke in a relative or friend, which could introduce selection bias.

## Conclusions

Our findings demonstrate the feasibility of EMDs identifying stroke symptoms such as side-specific arm paresis, side-directed CED, and aphasia via both telephone and video calls when using a standardized dispatch protocol that guides lay bystanders through stroke patient assessments. These examination steps enable the development of a focused, conditional aLVO screening process during emergency calls, paving the way for optimized dispatch strategies. The success of such protocols will likely depend on EMD training and the use of precisely formulated standardized instructions for laypersons.

## Supplementary Information

Below is the link to the electronic supplementary material.

Supplementary Material 1

## Data Availability

The datasets used and/or analysed during the current study are available from the corresponding author on reasonable request.

## Abbreviations

aLVO: Anterior circulation large-vessel occlusion
CED: Conjugated eye deviation
ECC: Emergency call center
EMD: Emergency medical dispatcher
FAST: Face Arm Speech Time
SEMD: Simulated emergency medical dispatcher
SP: Simulated patient
VC: Video call
