# Supplementary material for: Recognition of stroke symptoms indicative of anterior circulation large-vessel occlusion via telephone and video calls: a simulation study
Source: BMC Emerg Med. 2025 Sep 10;25:180. doi: 10.1186/s12873-025-01344-3 (PMC12424218; doi:10.1186/s12873-025-01344-3)

**SUPPLEMENT**

**Supplementary Table 1:** Simulated syndromes and symptoms (with corresponding NIHSS scores)

| **SP** | **Syndrome**  (total NIHSS) | **Motor arm** | **Best gaze** | **Best language** | **Dysarthria** | **Simulated calls** |
| --- | --- | --- | --- | --- | --- | --- |
| 1 | Severe MCA, left (22) | right arm: no effort against gravity (3) | forced deviation (2) to right | severe aphasia (2)* | severe dysarthria (2) | n = 11 |
| 2 | Moderate MCA, left (9) | right arm: drift (1) | normal (0) | severe aphasia (2)* | mild-to-moderate dysarthria (1) | n = 12 |
| 3 | Moderate pure motor stroke, left (4) | right arm: drift (1) | normal (0) | no aphasia (0) | mild-to-moderate dysarthria (1) | n = 12 |
| 4 | Severe pure motor stroke, left (9) | right arm: no movement (4) | normal (0) | no aphasia (0) | mild-to-moderate dysarthria (1) | n = 12 |
| 5 | Severe MCA, right (19) | left arm: no movement (4) | forced deviation (2) to left | no aphasia (0) | severe dysarthria (2) | n = 12 |
| 6 | Moderate MCA, right (8) | left arm: drift (1) | partial gaze palsy (1) to left | no aphasia (0) | mild-to-moderate dysarthria (1) | n = 13 |
| 7 | ACA,  right (5) | left arm: drift (1) | normal (0) | no aphasia (0) | normal (0) | n = 12 |
| 8 | Pure motor stroke, right (5) | left arm: some effort against gravity (2) | normal (0) | no aphasia (0) | normal (0) | n = 12 |

SP: simulated patient, NIHSS: National Institutes of Health Stroke Scale (points of each item in brackets). MCA: middle cerebral artery, ACA: anterior cerebral artery. *SPs presented severe aphasia with loss of speech fluency, fragmentary speech expression and reduced comprehension; they were only able to understand basic instructions.

**Supplementary Figure 1**: Protocol used by EMDs for the sequential detection of side of arm paresis, gaze deviation, and aphasia via VC


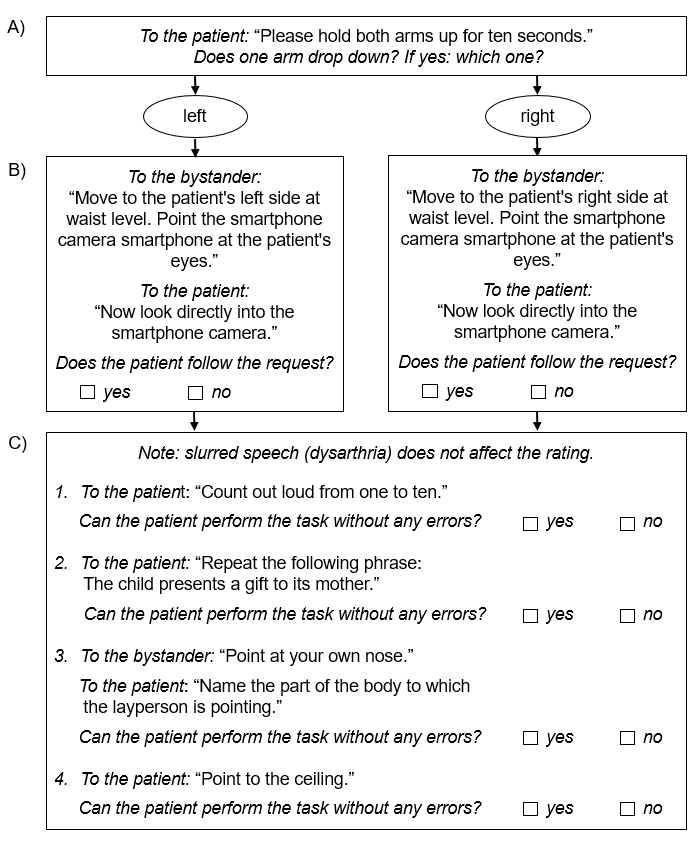

Supplement: Supplementary file 1 — Supplementary Material 1 [file 12873_2025_1344_MOESM1_ESM.docx]
